# Implementation of Nutrition Labels at the 2022 European Athletics Championships: An Observational Study of the Use and Perceptions of Athletes and Athlete Support Personnel

**DOI:** 10.3390/nu16244375

**Published:** 2024-12-19

**Authors:** Inês Maldonado, Catarina B. Oliveira, Pedro A. Branco, Mónica Sousa

**Affiliations:** 1 Nutrition and Lifestyle, NOVA Medical School, Faculdade de Ciências Médicas, NMS, FCM, Universidade NOVA de Lisboa, 1169-056 Lisboa, Portugal; 2 COD, Center of Sports Optimization, Sporting Clube de Portugal, 1501-806 Lisboa, Portugal; 3 CHRC, Comprehensive Health Research Center, NOVA Medical School, Faculdade de Ciências Médicas, NMS, FCM, Universidade NOVA de Lisboa, 1169-056 Lisboa, Portugal; 4 Medical & Anti-Doping Commission, European Athletics, 1003 Lausanne, Switzerland; pedro.branco@european-athletics.org; 5 CIDEFES, Sports, Physical Education, Exercise, and Health Research Center of Universidade Lusófona, Universidade Lusófona, 1749-024 Lisbon, Portugal

**Keywords:** catering, competition, dietary planning, nutrition labels, sport, sports nutrition, point-of-choice nutrition labels

## Abstract

Background/Objectives: Nutrition labels are an effective tool for providing nutrition information. Additionally, nutrient composition is one of the most dictating factors for athletes’ food choices; thus, we aimed to evaluate the use and perceptions regarding the nutrition labels implemented for the meals served at the 2022 European Athletics Championships (EAC). Methods: During mealtime at the team restaurants, participants completed an online self-administered questionnaire. We collected 280 questionnaires, 53.8% of the participants were male, most were athletes (78.9%), and 21.1% were athlete support personnel. Likert-type scales and open-ended questions were included to measure the labels’ importance, layout, influence on food choices, and participants’ understanding of the labels. Mann–Whitney and Kruskal–Wallis tests were used to compare answers. Results: Almost 40% of the participants used the nutrition labels occasionally (38.8%). Most participants were confident (41.9%) or moderately confident (31.3%) in making food choices because they had labels. Nutrition labels were considered important (41.0%) or very important (28.4%) by most participants, and 91.7% would like to have them in future championships. Athlete support personnel versus athletes (*p* = 0.037) and participants with dietary restrictions versus participants without (*p* = 0.028) were more confident in their food choices due to nutrition labels. Conclusions: Our results highlight that nutrition labels were helpful for both athletes and athlete support personnel during this EAC and that they should be maintained in future competitions.

## 1. Introduction

Athletes’ health and performance can be significantly affected by their nutritional status [1]. Thus, athletes’ food choices might be key during international competitions. During international competitions, the nutrient composition of the meals provided is one of the most dictating factors for athletes’ food choices [2]. Therefore, it is important to provide athletes with nutrition information regarding the meals they are provided.

Nutrition labels help sustain informed decisions and have proven effective in supporting athletes’ food choices during international competitions [3,4]. It has been reported that several athletes classify the provision of point-of-choice nutrition labels for menu items as important or very important [3]. Also, given the high proportion of athletes from different countries and sports who follow specialized dietary regimens [5], providing clear and informative nutrition labels in every sporting event’s dining hall is fundamental. Concerning nutrition labeling, some companies and governments developed front-of-pack (FOP) information systems, such as the Keyhole, Nutri-score, and Traffic Light, which are additional forms of expressing the nutrition declaration, and their efficacy has been studied [6,7,8]. Our data gathered during the 2019 European Athletics Under 20 and Under 23 Championships [9] agreed with what was reported in the literature, i.e., athletes expressed the need to have nutrition labels for the meals served during competitions. These results highlighted the importance of providing nutrition information for the food served at those events, namely a label with the name of the dish, the ingredient list, the nutritional composition, and information about allergens. In our study [9], the nutritional composition of the meals was among the factors with a greater contribution to food choice. Concerning nutrition label components, protein, carbohydrates, sugar, and energy were given greater importance [9].

Registered dietitians/nutritionists have been working alongside sports competition organizers, caterers, and other major international championships stakeholders to ensure that food provision meets international teams’ expectations [4,10,11,12,13], but there are no international guidelines that regulate food provision specifically for athletes and competition events. Considering that, in 2011, the European Athletics developed internal Nutritional Guidelines to standardize the menu proposals developed by the Local Organizing Committees for different competitions. By doing so, not only a greater level of satisfaction and organizational success could be achieved, but also a higher feeling of comfort and confidence by athletes and athlete support personnel regarding the food provided during the events. Over the years, the Nutritional Guidelines have been continuously improved and updated. Recently, considering that the provision of nutrition labels is mandatory at the Olympic Games and the Commonwealth Games [4], and based on the findings of our previous study [9], the European Athletics created a model of a point-of-choice nutrition label.

Nevertheless, nutrition labels are only helpful if they are fully understood by the recipients. Hence, we proposed to evaluate the athletes’ and athlete support personnel’s use and perceptions of point-of-choice nutrition labels implemented for the first time at the European Athletics Championships.

## 2. Materials and Methods

### 2.1. Type of Study

This observational study was conducted during the 2022 European Athletics Championships (from 13 August to 21 August) in Munich.

The study protocol was approved by the Ethical Committee of the Institutional Review Board (IRB) and by the European Athletics (March 2022) after full review, and written consent was obtained from all participants.

### 2.2. Participants and Procedures

All athletes and athlete support personnel who were at least 18 years old, participating at the 2022 European Athletics Championships, and having meals in the team restaurants were eligible to participate in this study. Data collection took place during mealtime at the teams’ restaurants. During these times, the research group approached the different teams to present the aim and the study protocol, as well as further information and explanations they might have considered relevant. After scanning a QR Code to access the questionnaire and giving their informed consent, participants completed the questionnaire. The completion of the questionnaire was performed at that moment in the presence of the researchers. Additionally, participants could, at any moment, refuse to participate in this study without any kind of penalties involved.

The final sample of 280 participants (athletes and athlete support personnel) comprised 14.4% (*n* = 221) of the 1540 athletes competing at the 2022 European Athletics Championships; participants’ sociodemographic characteristics are summarized in Table 1.

### 2.3. Instruments

For the purpose of this study, a short, self-administered online questionnaire was developed by the research team based on other studies [13,14] and the author’s experience, with the aim of evaluating the usefulness of the nutrition labels implemented at these European Athletics Championships. The questionnaire was available on an online platform (Easy Feedback) and included both closed- and open-ended questions. For most questions, five-point Likert-type scales were used. The frequency of nutrition label utilization was evaluated from 1 (never) to 5 (every time), the importance of providing the nutrition labels and relative importance of the different components of a nutrition label from 1 (not important) to 5 (very important), the amount of information displayed from 1 (insufficient) to 5 (excessive), the size of the nutrition labels from 1 (very small) to 5 (very big), the understanding of nutrition labels and the placement of nutrition labels from 1 (very poor) to 5 (very good), the usefulness of nutrition labels from 1 (not useful at all) to 5 (very useful), the confidence in making food/meal choices from 1 (not confident at all) to 5 (completely confident), and the likeliness of using nutrition labels to guide athletes’ meals ahead from 1 (extremely unlikely) to 5 (extremely likely). Open-ended questions were included to allow comments, suggestions, and requests.

### 2.4. Nutrition Label

The European Athletics created a model of nutrition labels to be used and adapted according to the meals provided. For the first implementation, the European Athletics considered that it would be advantageous for participants to have two formats of nutrition labels: a long and a short version. With this approach, two descriptions of the meals were provided: a full nutritional description, permanently available online and/or at the hotel welcome desks, and a concise version, visually more appealing and quicker to interpret, near the respective food item.

**Figure 1:**
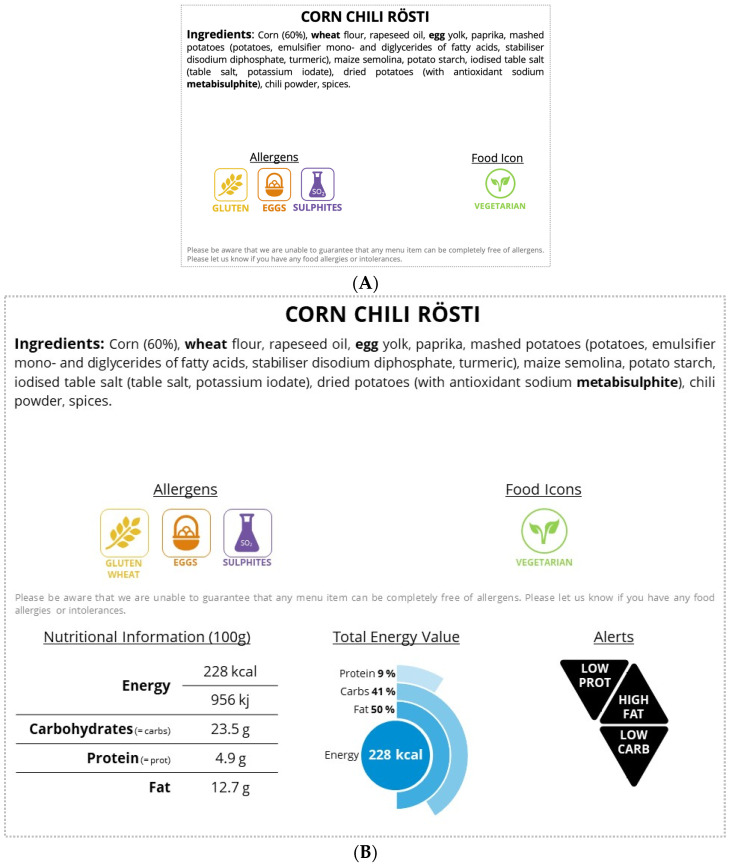
(**A**) Example of the short version of the point-of-choice nutrition labels. (**B**) Example of the long version of the point-of-choice nutrition labels.

For these championships, there were several team restaurants available, all serving the same menu previously approved by the European Athletics. The recipes were developed by one of the hotels (the leading hotel) and replicated by the rest. The nutrition information included in the labels was provided by the leading hotel. The short version label (Figure 1A) was available for all food items (except fruit) and included the list of ingredients, allergens, and food icons (identifying the types of meat, fish, and special dietary regimens). Regarding the long version label, the necessary information for its creation was only made available for vegetarian dishes. This version (Figure 1B) was placed at the welcome desk of team hotels for consulting and included the ingredient list, allergens icons, food icons, nutrition information per 100 g of recipe (with kcal and kj of energy, and grams of macronutrients), total energy graph (with kcal of energy and the percentage of the total energy value of macronutrients), and alerts qualitatively identifying the macronutrients amount (high carbohydrate; low carbohydrate; high fat; low fat; and high protein and/or low protein meals). The alerts were based on specific cutting points explained below.

The cutting points were based on the Regulation (EC) No 1924/2006 of the European Parliament and of the Council of 20 December 2006 [15] on the Traffic Light labeling system proposed by the British Government [16], on the Reference Values (DRV) established by the European Food Safety Agency (EFSA) [17], and on the Dietary Reference Intakes (DRI) of the Institute of Medicine (IOM) [18]. The words “carbohydrate” and “protein” were abbreviated into “carb” and “prot” due to constraints in label size and layout. Therefore, (i) “High fat” was used when the total fat content exceeded 8.4 g per 100 g of food, and “Low fat” when the total fat was no more than 3 g per 100 g of food; (ii) “High carb” was used when the total carbohydrate content accounted for more than 60% of the total energy value of the food, and “Low carb” when the total carbohydrate content accounted for less than 45% of the total energy value of the food; (iii) “High prot” was used when the total protein content accounted for more than 20% of the total energy value of the food, and “Low prot” when the total protein content accounted for less than 10% of the total energy value of the food.

### 2.5. Statistical Analysis

Data were entered and analyzed using the Statistical Package for Social Sciences (SPSS^®^) Statistical Software (version 26) [19].

The normality of the three continuous variables (age, the number of past international competitions attendance, and the years working with athletics) was checked using the Kolmogorov-Smirnov test; if normality was not assumed, the descriptive statistics were presented using the median, percentile 25, and percentile 75. Nominal or ordinal variables were described using frequencies. The nonparametric tests, Mann–Whitney test and Kruskal–Wallis test, were used to compare answers (i.e., differences between the answers of athletes and athlete support personnel, between male and female athletes, between athletes from different athletic disciplines, between the different categories of athlete support personnel, between participants with different education levels, between participants representing different countries, and between participants who had or did not have any dietary restrictions nor a dietary regimen prescribed by a nutritionist). Spearman correlation was used to describe the association between the frequency of nutrition label use and the importance given to nutrition labels. Statistical significance was set for *p* < 0.05. Countries were grouped into five regions (Northern Europe, Southern Europe, Western Europe, Eastern Europe, and Other countries, in accordance with the United Nations methodology [20].

Responses to open-ended questions were grouped according to topics for further analysis, and a representative example for each category was selected to exemplify open-ended answers.

## 3. Results

During the 2022 European Athletics Championships yield in Munich, we collected 280 valid surveys, 19 of which were excluded due to being incomplete. The majority of the participants (78.9%) were athletes, with the remainder being athlete support personnel (21.1%). Athletes’ median age was 26 (23; 39) years, and athlete support personnel’s was 42 (35; 52) years. The sociodemographic characteristics of the participants are summarized in Table 1. Most participants were representing Western (30.8%) and Southern (29.0%) European countries. Detailed information about the countries that participants were representing is available as a Supplementary File. Regarding athletes, the median of past internationalizations was five (3; 10), and regarding athlete support personnel, the median of the years working in athletics was 16 (9; 29).

Most participants did not follow a dietary regimen prescribed by a dietitian/nutritionist (73.9%) nor had dietary restrictions (79.9%). Among those with dietary restrictions (20.1%), dairy products, lactose intolerance, or both, were the most frequently reported (3.6%, *n* = 9), followed by vegan or vegetarian restrictions (3.2%, *n* = 8), and gluten-free (2%, *n* = 5).

### 3.1. Nutrition Label Use

Our data show that the median nutrition label utilization was occasional (38.3%), with 5.7% of the participants using it always, 14.6% almost every time, 13.8% almost never, and 25.3% of the participants never using it. We did not find differences between the answers of male and female participants (*p* = 0.251) nor between athletes and athlete support personnel (*p* = 0.366). Among athletes from different events, we did not find differences regarding nutrition label use between all groups (*p* = 0.291), among different categories of athlete support personnel (*p* = 0.257), nor according to the different levels of education (*p* = 0.266). However, we found that participants who followed a prescribed dietary regimen used the nutrition labels more often (*p* = 0.002, 16.4% never; 9.8% almost never; 37.7% occasionally; 21.3% almost every time; 14.8% every time) compared to participants who did not (28.1% never; 15.1% almost never; 38.5% occasionally; 14.6% almost every time; 3.6% every time). Depending on the country region, nutrition labels were used differently. Southern European countries used nutrition labels more often (15.8% never; 10.5% almost never; 40.8% occasionally; 27.6% almost every time; 5.3% every time) compared with Western countries (*p* = 0.04, 39.5% never; 9.9% almost never; 32.1% occasionally; 12.3% almost every time; 6.2% every time) and comparing with Northern countries (*p* < 0.001, 28.9% never; 26.3% almost never; 39.5% occasionally; 2.6% almost every time; 2.6% every time). The most frequent explanations referred by participants to not use nutrition labels more often were reasons related to not needing to, preferring only to see the food, and not having food allergies or intolerances (Table 2).

Nevertheless, nutrition labels were considered important (41.0%) or very important (28.4%) by most participants, and we did not find any differences between the answers of male and female participants (*p* = 0.509), athlete and athlete support personnel (*p* = 0.401), following or not a prescribed regimen by a nutritionist (*p* = 0.129), having or not any dietary restrictions (*p* = 0.127), between athletes from different events (*p* = 0.431), between participants with different education levels (*p* = 0.680), nor between the different categories of athlete support personnel (*p* = 0.278), nor depending on country region (*p* = 0.131). We found a weak but significant correlation between the frequency of nutrition label use and the importance given to nutrition labels (ρ = 0.319; *p* < 0.001).

### 3.2. Nutrition Label Content

**Figure 2:**
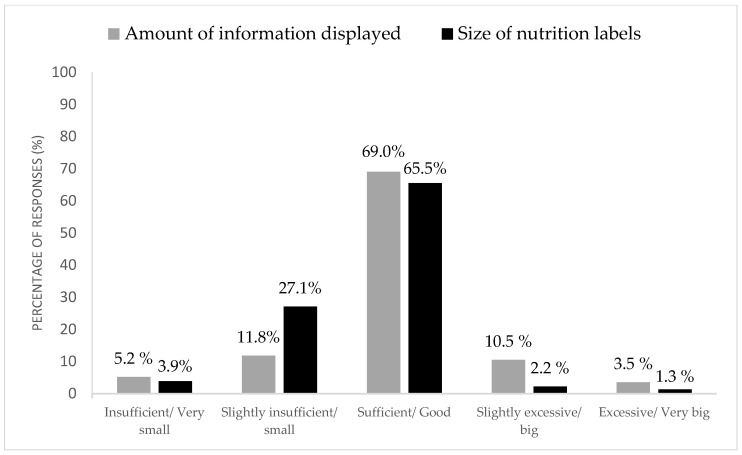
Short version nutrition label amount of information displayed and size.

Concerning the design of the short version nutrition labels, the majority of the participants considered sufficient (69.0%) the amount of information displayed (Figure 2), and most participants (65.5%) rated the size of the nutrition label as good. Still, participants suggested that some items could be added to the short version of nutrition labels, with some examples in Table 3. Additionally, the placement of the labels was considered good.

Regarding the long version labels, only 26.5% of the participants saw them, but similarly to the short version label, the amount of information displayed was considered sufficient (65.8%) by most participants, who also rated their understanding of the labels as good (61.6%). Even though most participants did not see this label, its placement was considered good (57.5%).

**Figure 3:**
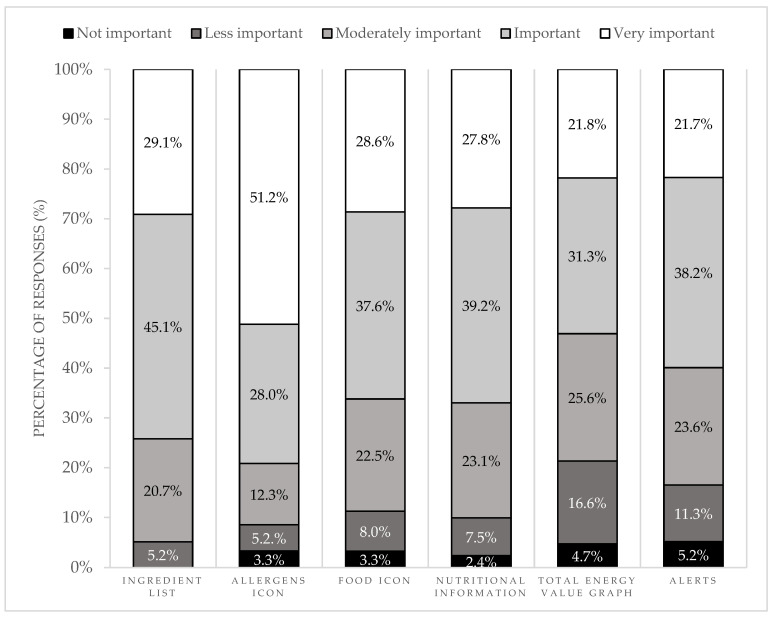
Importance given to the long version of nutrition label components.

From all nutrition label components, allergens information was the one to which participants attached greater importance, followed by the ingredient list (Figure 3).

A higher percentage of athletes with a dietary regimen prescribed by a registered dietitian considered nutrition information on the labels very important (38.8%) compared to athletes without a prescribed dietary regimen (23.5%, *p* = 0.024). A higher percentage of athletes with dietary restrictions considered food icons on the labels very important (45.7%) compared to athletes without dietary restrictions (23.4%, *p* = 0.005). No differences were found on the answers of participants from different categories (ingredient list, *p* = 0.897; allergens icon, *p* = 0.274; food icons, *p* = 0.459; nutritional information, *p* = 0.806; total energy value graph, *p* = 0.093; alerts, *p* = 0.320), between participants with different education levels (ingredient list, *p* = 0.468; allergens icon, *p* = 0.927; food icons, *p* = 0.981; nutritional information, *p* = 0.328; total energy value graph, *p* = 0.810; alerts *p* = 0.539), nor between athletes from different events (ingredient list, *p* = 0.819; allergens icon, *p* = 0.244; food icons, *p* = 0.878; nutritional information, *p* = 0.627; total energy value graph, *p* = 0.912; alerts, *p* = 0.714).

### 3.3. Nutrition Label Utility

**Figure 4:**
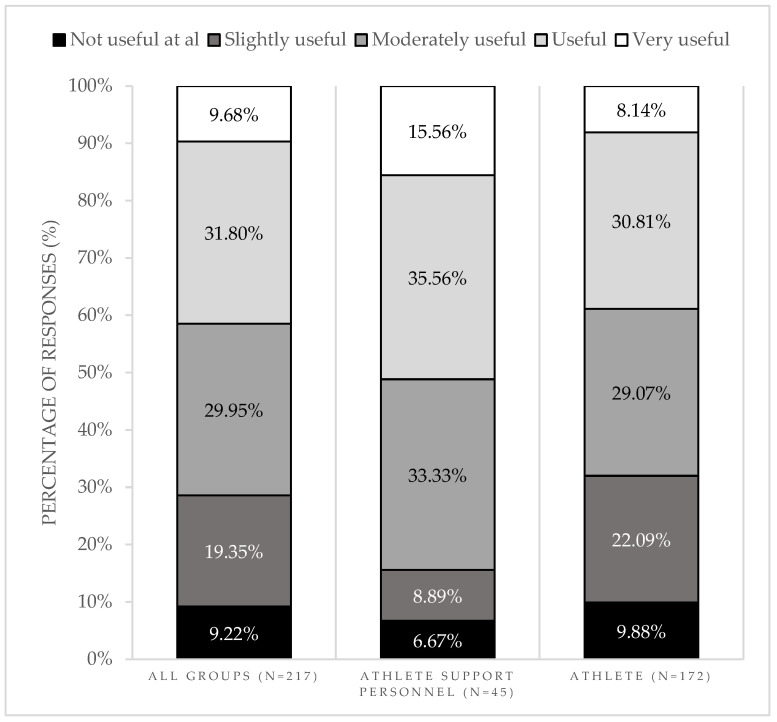
Usefulness of nutrition labels in assisting participants to find meals by the type of participant.

Most participants considered nutrition labels useful (31.8%) or moderately useful (30%) to find the foods they needed. We did not find any differences between the answers of (i) male and female participants (*p* = 0.634); (ii) participants following or not a prescribed regimen by a nutritionist (*p* = 0.113); (iii) having or not any dietary restrictions (*p* = 0.104); (iv) athletes from different events (*p* = 0.275); (v) participants with different education levels (*p* = 0.634); and (vi) different activities as athlete support personnel (*p* = 0.318). However, we found that athlete support personnel considered nutrition labels to be more useful compared with athletes (*p* = 0.036) (Figure 4).

**Figure 5:**
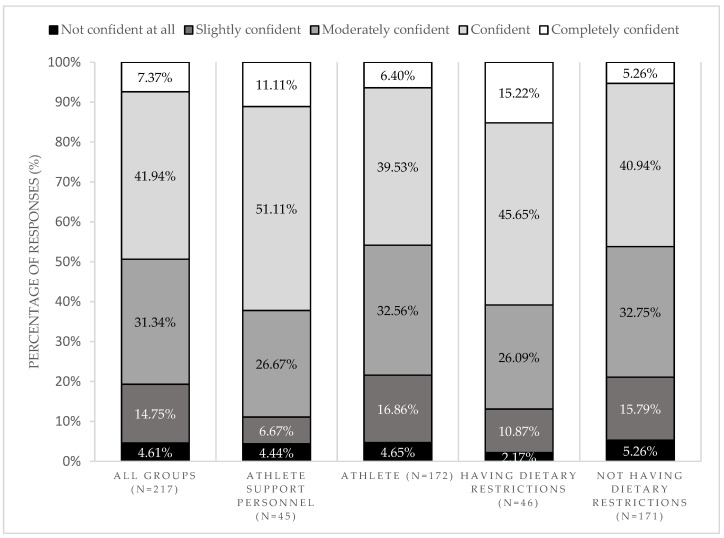
Confidence in making food choices because they had nutrition labels, by the type of participant.

Regarding the influence of nutrition labels on food choice, 27.6% (*n* = 60) of the participants changed their initial food choices after reading the label. Some of the reasons given by the participants were related to the presence of allergens or other food ingredients, which they aimed to restrict from their diet (*n* = 8). Additionally, most participants were confident (41.9%) or moderately confident (31.3%) in making food choices because they had nutrition labels. We found differences in the answers regarding the reasons for feeling confident in food choices due to the presence of nutrition labels between the athlete support personnel and athletes (*p* = 0.037) and between participants with or without dietary restrictions (*p* = 0.028) (Figure 5). Additionally, most athlete support personnel considered that nutrition labels were important (52.2%) or very important (30.4%) for athletes, and they were likely (52.2%) to use the nutrition labels to guide the athletes’ meals ahead.

Most participants (91.7%) would like to have nutrition labels in future championships.

## 4. Discussion

This research provided the opportunity to study the nutrition label use and opinion of both athletes and athlete support personnel in a particular environment—the 2022 European Athletics Championships. We found that in a sports context, the presence of nutrition labels helped not only athletes but also other team members, such as coaches, physiotherapists, doctors, and other staff members, with their food choices, regardless of their activity, age, gender, or level of education. Given the prevalence of participants with some dietary restriction or following a prescribed dietary plan and taking into account both the frequency of nutrition label utilization and the results from the influence of nutrition labels on food choice, there is no doubt that nutrition labels play a crucial role in assisting food choice during an international competition.

### 4.1. Nutrition Label Use and Opinion Based on Sex and Country

Women typically report a higher frequency of nutrition label use compared to men [5,21,22]. However, among athletes, this difference seems to be diluted. In accordance with our findings, a previous study with athletes [14] found no differences in nutrition label use between sexes; nevertheless, the authors still found that female athletes attached greater importance to providing nutrition labels, compared to men, differently from what we found: no differences according to sex regarding this matter. Additionally, in a study that evaluated the use and understanding of nutrition labels in the general population [22], female participants attached greater importance to fiber, sugar, and carbohydrates. In our study, we did not evaluate the importance given specifically to these items, but we assessed the relative importance of the ingredient list, allergens, food icons, nutrition information, total energy value, and alerts. In a previous study [14], female athletes were the ones who gave the most suggestions for the improvement of nutrition labels related to the ingredient list and more specific serving size information, but in our results, we did not find differences in the importance given to the different items of the nutrition label between sex, similarly to what we found in a previous study with younger European athletes [9]. A possible explanation is related to the particularities of the athletic population, and their nutrition knowledge and concerns, despite the sex.

A possible explanation for why nutrition labels were significantly more used by participants from Southern countries versus Northern countries is that FOP nutrition labels, specifically the Keyhole, were adopted in Sweden in 1989, and other northern countries (Denmark, Iceland, Norway, and Lithuania adopted this FOP label) [23]. In contrast, southern countries such as Belgium and Spain only adopted the Nutri-Score FOP nutrition label after 2017 [23]. Thus, having an FOP nutrition label may help consumers make food choices based only on this symbol and not being used to interpret other nutritional information.

### 4.2. Nutrition Label Use and Opinion Based on the Type of Participant

Athlete support personnel considered nutrition labels useful for athletes; additionally, this group of participants considered nutrition labels to be more useful compared with athletes. These are important data, in line with what was described in recent research [24] where sports technical team members had better nutrition knowledge than athletes. That same study [24] also concluded that sport technical team members tended to underestimate their own nutrition knowledge, and this may indicate that coaches, medical doctors, physiotherapists, massage therapists, and other athlete support personnel value the role of nutrition and dietary practices during a highly competitive period. In a previous study that collected the point of view of nutrition experts during the 2012 Olympic Games [13], a higher percentage of participants rated allergens information as being important compared to the alerts regarding the nutritional content; in another study [14], athletes, however, attributed greater importance to nutrients comparing to allergens. In our study, we could not find differences regarding the importance that was given by athletes or athletes’ support personnel to different nutrition label components, but consistently, our results show the great importance that both allergens and nutrition information have for the sports population.

### 4.3. Nutrition Label Use and Opinion Based on Having a Prescribed Diet or a Dietary Restriction

In a recent study [22], authors found that participants who followed a diet used nutrition labels more often and valued the nutrition information displayed on the labels more, which is in accordance with our results.

In our study, 20.1% of the participants had some kind of dietary restriction, and 26.1% followed a dietary regimen prescribed by a nutritionist; this prevalence of food avoidance is slightly higher compared to our data from the 2019 European Athletics Under 20 and Under 23 Championships [9], where 13% of the participants reported having a food avoidance. Nevertheless, our results are below the data from the 2010 Commonwealth Games [25], where the authors reported that 21% of the participants avoided meat and other animal products, 18% had another type of food allergies and intolerances, and 7% of food avoidances were related to religion. In our study, milk, dairy, lactose, gluten, and avoiding animal products were the most frequently reported food restrictions. Participants with dietary restrictions, compared to the ones without, valued the presence of the allergens on the label more. The EU Regulation No 1169/2011 [26], which establishes the general principles and requirements of food labeling with the objective of protecting consumers’ health and interests, states that any ingredient from the Annex II of the EU Regulation No 1169/2011 causing allergies or intolerances used in the manufacture or preparation of food, should be obligatorily mentioned. Therefore, caterers must always specify the allergens present in meals [26]. Thus, the nutrition labels provided during international competitions should always include this information.

### 4.4. Nutrition Labels Positioning, Elements, and Layout

In addition to the allergens and the list of ingredients, participants valued nutrition information, as seen in the past [9]. Additionally, participants gave suggestions for improvement, reinforcing the need for more detailed energy and macronutrient information. These data may be explained by the fact that for this study, the nutritional composition of the meals was described on the large version of the labels, which were only available for certain foods and displayed at the welcome desks of the hotels. Hence, since the placement of the short version labels was considered good, we propose a new and single version of labels in future championships that should be displayed near each food item. This new label is intended to be a combined and improved version of the two existing labels.

### 4.5. Future Directions

Layout design and characteristics (e.g., color, font and font size, label size, and label placement) may be decisive for the label to be processed by those under time constraints, and less complex labels require less attention to be processed [21]. Thus, it is necessary to plan and test which information should be presented, as performed in this study; moreover, an appealing and simple label should be provided. Considering the feedback received from participants about the short version label, we propose that only a single short model be displayed at the forthcoming European Athletics Championships. This label shall include the name of the food, the list of ingredients, the allergens information, the icons identifying special dietary regimens, and the nutritional composition, and it should be positioned next to the food. Considering that some participants asked for icons or figures identifying ingredients or food types, we propose keeping the allergen icons, which are colorful and enhance labels appealing, and the icons identifying special dietary regimens and foods. Additionally, we propose to include the nutrition declaration (per 100 g or 100 mL of food product) to answer participants’ demands.

Regarding the alerts stating for high/low carbohydrates, protein, or fat, they are in accordance with the Regulation (EC) No 1924/2006 of the European Parliament and of the Council of 20 December 2006 [15], and with the traffic light, a type of Front-of-Pack (FOP) nutrition labeling from the British Government [16], and were adjusted to better suit the athletic population. However, it is important to acknowledge that they, as other FOP nutrition labels, are an additional form of expressing the nutrition declaration [8]. Therefore, considering participants’ opinions on this nutrition label component and the limited space for a nutrition label near the food items, we propose not to include these alerts in future labels to be provided in European athletics competitions.

In the future, the promotion of food literacy programs among athletes and athlete support personnel seems relevant. In addition, comparing the nutrition knowledge of this population based on the country region might be pertinent.

The use of Artificial Intelligence in biomedical sciences, including food and nutrition [27,28,29], has been widely developed in recent years. Image-based food recognition systems (systems that combine the use of a mobile device camera with computer vision methods) are being used to support dietary assessment [30]. Hence, an alternative solution that embraces the development of smartphone apps [13] with Artificial Intelligence solutions might be a valuable tool to provide both athletes and athlete support personnel with all the information they need about the meals served in a sustainable, accessible, and convenient setup.

Our study has some limitations that should be recognized. Firstly, there might have been language-related concerns since the questionnaire was in English, and this may have excluded some potential non-English-speaking participants, and miscomprehension errors may have occurred. Nevertheless, researchers were present while participants completed the questionnaire to clarify any necessary information. In addition, participants were frequently sited next to peers, which may have influenced their responses. Finally, a convenience sample was recruited; consequently, it may not represent all the participants of these European Athletics Championships. Nevertheless, although we were only able to include 280 participants, we collected answers from elite-level athletes and athlete support personnel from 35 nationalities.

## 5. Conclusions

In conclusion, our study collected a singular perspective from European athletics teams during an elite competitive environment. Nutrition labels proved to be useful both for athletes and for athlete support personnel. Given our results, and in order for future labels to be better adjusted to participants’ needs and still be attractive and easy to interpret, we suggest developing only one version of the nutrition label. This new label should include the name of the dish, a list of ingredients, allergens icons, icons identifying special dietary regimens and foods, and its nutritional composition. In future championships, it is important to ensure that these labels are available for all foods. Additionally, future research is needed regarding nutrition labels provided in other international competitions that include other sports. Moreover, research on the provision of nutrition labels combined with the use of Artificial Intelligence vision and product recognition technologies would be interesting.

## Figures and Tables

**Table 1:** Participants sociodemographic characteristics.

	Athlete (*n* = 221)	Athlete Support Personnel (*n* = 59)	Total (*n* = 280)
Sex	% (*n*)	% (*n*)	% (*n*)
Male	42.2 (102)	79.7 (47)	53.8 (149)
Female	53.8 (119)	20.3 (12)	46.8 (131)
Country region	% (*n*)	% (*n*)	% (*n*)
Eastern Europe ^a^	18.2 (40)	22.0 (13)	19.0 (53)
Northern Europe ^b^	15.5 (34)	18.6 (11)	16.1 (45)
Southern Europe ^c^	29.1 (64)	28.8 (17)	29.0 (81)
Western Europe ^d^	30.9 (68)	30.5 (18)	30.8 (86)
Other countries ^e^	6.4 (14)	-	5.0 (14)
Event of athletics	% (*n*)		% (*n*)
Sprints ^f^	39.3 (84)		39.3 (84)
Middle distance ^g^	19.6 (42)		19.6 (42)
Distance ^h^	17.8 (38)		17.8 (38)
Jumps ^i^	10.3 (22)		10.3 (22)
Throws ^j^	9.8 (21)		9.8 (21)
Combined events ^k^	3.3 (7)		3.3 (7)
Activity as athlete support personnel		% (*n*)	% (*n*)
Coach		63.8 (37)	63.8 (37)
Medical doctor		8.6 (5)	8.6 (5)
Physiotherapist		15.5 (9)	15.5 (9)
Massage therapist		3.4 (2)	3.4 (2)
Other		8.6 (5)	8.6 (5)
Level of education	% (*n*)	% (*n*)	% (*n*)
Completed university or other tertiary institution	61.5 (136)	71.2 (42)	63.6 (178)
Completed high school	32.6 (72)	23.7 (14)	30.7 (86)
Completed intermediate/middle school	4.1 (9)	5.1 (3)	4.3 (12)
Completed primary school	0.5 (1)		0.4 (1)
Never attended school	1.4 (3)		1.1 (3)

^a^—Bulgaria, Czechia, Great Britain, Poland, and Ukraine; ^b^—Denmark, Finland, Iceland, Ireland, Latvia, Lithuania, and Sweden; ^c^—Albania, Andorra, Bosnia and Herzegovina, Croatia, Gibraltar, Greece, Italy, Macedonia, Montenegro, Portugal, Serbia, Slovenia, and Spain; ^d^—Austria, Belgium, France, Germany, Netherlands, and Switzerland; ^e^—Cyprus, Israel, and Turkey; ^f^—100, 200, 400, 100, or 110 m hurdles, 400 m hurdles, 4 × 100 m, and 4 × 400 m; ^g^—800 m, 1500, 3000, 5000, and 3000 m steeplechase; ^h^—high jump, pole vault, long jump, and triple jump; ^i^—shot put, discus throw, hammer throw, and javelin throw; ^j^—10,000 m and 10 or 20 km race walk; ^k^—heptathlon, decathlon.

**Table 2:** Reasons named by participants to not use the nutrition labels more often.

Response Category	Number of Answers (*n*)	Example of Responses
Not needing to	17	*Not needed.*
Preferring to see the food	8	*I prefer to see what the food is, rather than its nutritional content.*
Not having food intolerances nor food allergies	7	*No allergies or special diet.*
Not having interest on it	7	*No interest.*
Not knowing the reason	6	*No particular reason.*
Not understanding or not having seen the label or not knowing about the existence of the labels	6	*I just did not know the existence of them.*
Preferring to see the name of the dish	2	*I look for the name of the dish and that is it.*

**Table 3:** Items to be added to the short version nutrition labels suggested by participants.

Response Category	Number of Answers (*n*)	Example of Responses
Nutrition information	16	*You need to add nutrition information regarding salt, fat, saturated fat, protein,* etc.
Layout	5	*Just make the labels a bit bigger to be more likely to read/see*
Figures/photos	2	*Adding photos of the ingredients*
Alerts	5	*Include rating: High/low in protein/fat/carbs/(sugar)*
Other	16	*More languages*

## Data Availability

The data are not publicly available due to privacy and ethical restrictions.

## Supplementary Materials

The following supporting information can be downloaded at https://www.mdpi.com/article/10.3390/nu16244375/s1: Supplementary Table: Participants’ detailed information about the countries they represented.
