# Supplementary material for: Implementation of Nutrition Labels at the 2022 European Athletics Championships: An Observational Study of the Use and Perceptions of Athletes and Athlete Support Personnel
Source: Nutrients. 2024 Dec 19;16(24):4375. doi: 10.3390/nu16244375 (PMC11677057; doi:10.3390/nu16244375)
Supplement: Supplementary file 1 [file nutrients-16-04375-s001.zip › nutrients-3356537-supplementary.pdf]

	Athlete (n=221)	Athlete support personnel (n=59)	Total (n=280)
Albania	0.5 (1)		0.4 (1)
Austria	0.9 (2)		0.7 (2)
Belgium	9.5 (21)	15.3 (9)	10.7 (30)
Bulgaria	0.5 (1)	1.7 (1)	0.7 (2)
Croatia	0.5 (1)		0.4 (1)
Cyprus	1.4 (3)		1.1 (3)
Czech Republic	2.7 (6)		2.1 (6)
Denmark	3.2 (7)		2.5 (7)
Finland	5.9 (13)	6.8 (4)	6.1 (17)
France	10.9 (24)	6.8 (4)	10.0 (28)
Germany	0.9 (2)	3.4 (2)	1.4 (4)
Gibraltar	0.9 (2)	1.7 (1)	1.1 (3)
Great Britain	9.5 (21)	15.3 (9)	10.7 (30)
Greece	2.3 (5)	1.7 (1)	2.1 (6)
Hungary		5.1 (3)	1.1 (3)
Iceland	0.9 (2)		0.7 (2)
Ireland	1.8 (4)	3.4 (2)	2.1 (6)
Israel	2.7 (6)		2.1 (6)
Italy	5.4 (12)	3.4 (2)	5.0 (14)
Latvia	0.9 (2)		0.7 (2)
Lithuania	0.9 (2)		0.7 (2)
Luxembourg		1.7 (1)	0.4 (1)
Macedonia	0.5 (1)	1.7 (1)	0.7 (2)
Montenegro	0.5 (1)		0.4 (1)
Netherlands	5.4 (12)	3.4 (2)	5.0 (14)
Norway		5.1 (3)	1.1 (3)
Poland	4.5 (10)		3.6 (10)
Portugal	10.0 (22)	15.3 (9)	11.1 (31)
Serbia	1.4 (3)	1.7 (1)	1.4 (4)
Slovenia	0.9 (2)		0.7 (2)
Spain	6.3 (14)	3.4 (2)	5.7 (16)
Sweden	1.8 (4)	3.4 (2)	2.1 (6)
Switzerland	3.2 (7)		2.5 (7)
Turkey	2.3 (5)		1.8 (5)
Ukraine	0.9 (2)		0.7 (2)

Supplementary table. Participants detailed information about the countries they represented
